# Convolutional Neural Network–Vision Transformer Architecture with Gated Control Mechanism and Multi-Scale Fusion for Enhanced Pulmonary Disease Classification

**DOI:** 10.3390/diagnostics14242790

**Published:** 2024-12-12

**Authors:** Okpala Chibuike, Xiaopeng Yang

**Affiliations:** 1 Department of Human Ecology & Technology, Handong Global University, Pohang 37554, Republic of Korea; myokpala@handong.ac.kr; 2 School of Global Entrepreneurship and Information Communication Technology, Handong Global University, Pohang 37554, Republic of Korea

**Keywords:** vision transformer, convolutional neural network, gated control mechanism, multi-scale fusion module, pulmonary diseases

## Abstract

Background/Objectives: Vision Transformers (ViTs) and convolutional neural networks (CNNs) have demonstrated remarkable performances in image classification, especially in the domain of medical imaging analysis. However, ViTs struggle to capture high-frequency components of images, which are critical in identifying fine-grained patterns, while CNNs have difficulties in capturing long-range dependencies due to their local receptive fields, which makes it difficult to fully capture the spatial relationship across lung regions. Methods: In this paper, we proposed a hybrid architecture that integrates ViTs and CNNs within a modular component block(s) to leverage both local feature extraction and global context capture. In each component block, the CNN is used to extract the local features, which are then passed through the ViT to capture the global dependencies. We implemented a gated attention mechanism that combines the channel-, spatial-, and element-wise attention to selectively emphasize the important features, thereby enhancing overall feature representation. Furthermore, we incorporated a multi-scale fusion module (MSFM) in the proposed framework to fuse the features at different scales for more comprehensive feature representation. Results: Our proposed model achieved an accuracy of 99.50% in the classification of four pulmonary conditions. Conclusions: Through extensive experiments and ablation studies, we demonstrated the effectiveness of our approach in improving the medical image classification performance, while achieving good calibration results. This hybrid approach offers a promising framework for reliable and accurate disease diagnosis in medical imaging.

## 1. Introduction

In recent years, global health has witnessed a notable increase in the prevalence of lung and respiratory-related conditions, affecting a significant proportion of the population. These conditions pose a substantial challenge, necessitating the swift and precise detection and diagnosis of various lung diseases, such as Pneumonia, COVID-19, and tuberculosis (TB). The timely administration of effective treatment is crucial, making the use of advanced diagnostic tools essential [1]. Pulmonary diseases are particularly challenging to detect and diagnose in the early stages due to their deceptive initial symptoms, requiring rigorous and often extensive diagnostic procedures [2]. The accurate diagnosis of pulmonary diseases in the early stages is vital for patient management and care, yet the process is often complicated and prone to errors. The World Health Organization (WHO) recommends the use of chest radiography, such as chest X-ray (CXR), magnetic resonance imaging (MRI), and computed tomography (CT) images, as the principal modalities for diagnosing and screening these diseases because of their notable sensitivity [3]. However, interpreting these images is labor-intensive, subject to individual bias, lacks specificity, and is prone to misdiagnosis due to similarities in the radiologic patterns among various lung diseases [2].

To address these challenges, AI-based computer-aided diagnosis (CAD) systems have been developed to automatically diagnose and detect pulmonary diseases using chest radiography [4]. These CAD systems employ advanced deep learning models, such as convolutional neural networks (CNNs), recurrent neural networks (RNNs), and transformers, in the analysis, segmentation, and classification of pulmonary diseases.

The emergence of deep learning models marks a transformative period in the field of medical image analysis, holding considerable promise in the detection of pulmonary and other diseases [5]. These models make medical imaging analysis faster and easier, offering a promising avenue for improving the accuracy and efficiency of pulmonary disease detection [6]. However, CNNs and transformer models have their inherent limitations. CNNs are limited in capturing long-range dependencies [7], while transformer models, such as Vision Transformers (ViTs) [8], have a limited capability of capturing low-level features and are data-hungry [9]. Recent surveys [9,10,11] on hybrid CNN-ViT architectures, such as ResNet-ViT and UNet-ViT, highlighted the growing importance of hybrid architectures that combined the strengths of CNNs and ViTs to address these limitations. These hybrid vision transformers (HVTs) integrated CNN layers to capture local features, while leveraging the transformers’ ability to learn long-range dependencies, thereby providing more comprehensive feature representation. HVTs have demonstrated significant potential in computer vision, especially in medical imaging tasks, including segmentation and classification, by overcoming challenges such as data inefficiency and a lack of image-related inductive bias [10,11] through incorporating both the global and local contexts. However, the existing models mostly integrated ViTs with a single CNN model, limiting the sufficient extraction of various features from medical images.

To overcome these limitations, we proposed a gated hybrid framework that fuses the feature extraction capabilities of multiple component blocks consisting of CNN and ViT encoder architectures. In the framework, CNN is used to extract the local features, while the ViT is used to extract the long-range dependencies. Different component blocks use different CNN models to sufficiently extract various features from medical images. Then, the significant features are selectively extracted through a gated control mechanism and are then fused with a multi-scale fusion module for more comprehensive feature representation. The main contributions of this study are outlined as follows:

- We propose a hybrid architecture that allows for the integration of any CNN and ViT encoder within component blocks. Each component block is carefully designed to capture the low-range and long-range dependencies effectively.
- We introduce a gated attention control mechanism, which selectively emphasizes the important features through channel-, spatial-, and element-wise attention. This mechanism modulates and refines the feature representations by dynamically controlling the flow of relevant information.
- We present a multi-scale fusion module that captures single-level and multi-level features. This module uses an Inception-style design to combine fine-grained, medium-scale, and large-scale features across multiple branches, ensuring more comprehensive feature representation.

## 2. Related Work

### 2.1. Pulmonary Disease Detection Based on CNN Architecture

Mousavi et al. [12] proposed a COVID-19 detection framework using respiratory sounds (coughing) and medical images using the Internet of Health Things (IoHTs). The authors employed two datasets of CXR and CT images to fine-tune pre-trained Incep-tionResNetV2, InceptionV3, and EfficientNetB4 models for three-class classification tasks. Rajaraman and Antani [13] introduced a modality-specific deep learning model ensemble to enhance TB detection using CXR images. The authors combined custom-built CNNs and pre-trained CNNs to learn the modality-specific features. The predictions from the best-performing models were combined using ensemble methods, which improved the classification performance compared with an individual model. Vinayakumar et al. [14] proposed a multichannel ensemble framework using EfficientNet-based models (EfficientNetB0, EfficientNetB1, and EfficientNetB2) to extract features. These features were then fused and passed into a stacked ensemble learning classifier, enhancing the overall classification performance. Sasikaladevi and Revathi [15] developed a custom deep learning framework for the early detection and prognosis of TB from CXR images. Their framework employed a deep Fused Linear Triangulation (FLT) approach to handle intraclass variation and interclass similarities, accurately visualizing the infected regions in CXR images without requiring segmentation. Urooj et al. [16] presented a stochastic learning-based Artificial Neural Network (ANN) model using CXR images to detect TB. The method introduced random variations into the network by assigning stochastic transfer functions or weights, effectively detecting abnormalities in the CXR images across various levels of TB complexity. While conventional CNN-based models effectively capture local features, they lack the mechanisms for emphasizing relevant information or integrating multi-scaled features. None of these models incorporated a gated attention control mechanism or a multi-scale fusion module, which limited their capabilities in prioritizing significant features or combining fine-grained, medium-scale, and large-scale features.

### 2.2. Pulmonary Disease Detection Based on Transformer Architecture

Mabrouk et al. [17] proposed an ensemble learning method for Pneumonia detection in CXR images, leveraging three pre-trained models: DenseNet169, MobileNetV2, and Vision Transformer (ViT). The authors argued that combining the features from these models using a probability-based ensemble approach significantly enhanced the classification performance. The ensemble method used by the authors inherently increased the computational complexity during inference, which could limit its deployment in resource-constrained environments. Sun et al. [18] introduced a convolutional transformer model for lung disease classification based on CXR images. They modified the transformer encoder’s attention mechanism by replacing it with an axial attention module and assigning a position offset term, resulting in an improved classification performance. Though the model demonstrated its effectiveness on a small dataset, the model lacked generalizability to more diverse datasets. Real-world medical imaging datasets often include a broader spectrum of conditions, varying image quality, and class imbalance. The model’s effectiveness in such scenarios remains untested, which limits its clinical applicability. Ukwuoma et al. [19] proposed a ViT-based model for lung disease classification in CXR images. The model used an ensemble technique to derive features, followed by global second-order pooling to extract the higher-order global features. This approach combined deep feature extraction with global feature representation for enhanced classification accuracy. The combination of multiple features from different CNN models using an ensemble approach could potentially enhance the model’s performance by leveraging diverse feature representation. However, there is no mechanism or control (gate) to filter or prioritize the relevant features during the concatenation process. This lack of feature relevance control could result in the inclusion of less-relevant or redundant features, thereby limiting the model’s overall performance. Moreover, the indiscriminate concatenation of features increased the dimensionality of the feature space, potentially introducing noise and making the model more prone to overfitting, especially on a small dataset. Ren et al. [20] introduced the ResNet-50 merged transformer (RMT-Net), a model combining ResNet-50 with ViT architecture. This approach aimed to leverage the CNN’s ability to extract local features and the transformer’s capability to capture long-range features, thereby reducing the computational cost and accelerating detection. The RMT-Net featured a four-stage block design, with global self-attention applied in the first three stages and residual blocks in the fourth stage for feature extraction. While this approach successfully captured both the global and local features, a significant limitation was the absence of adaptive feature selection mechanisms. The model did not incorporate strategies, such as attention gating or feature pruning, to control the features prioritized for classification. As a result, irrelevant or redundant features might be involved in the decision-making process, potentially degrading the model’s generalization and performance on unseen datasets.

### 2.3. Gated Mechanisms 

Gated mechanisms facilitate easier gradient back-propagation through depth or time [21]. The primary idea behind a gating module is to control information flow based on the learned parameters, prioritizing the most relevant features for subsequent layers. Zhang et al. [22] introduced a framework for palmprint recognition that integrates CNNs and a transformer, leveraging the local extraction capabilities of CNNs and the global modeling strengths of transformers. This framework includes a gating mechanism and an adaptive feature fusion module, which filter and integrate the features extracted by the backbone network, ensuring a robust palmprint feature extraction and recognition performance. Schlemper et al. [23] proposed an attention gate (AG) model for medical image analysis designed to focus on target structures of varying shapes and sizes. AGs automatically suppress the irrelevant regions and highlight the salient features without requiring explicit tissue or organ localization modules. This mechanism can be seamlessly integrated into standard CNNs like VGG or U-Net, enhancing the model’s sensitivity and prediction accuracy with a minimal computational overhead. Fang and Han [24] developed an attention-modulated network based on the U-Net architecture, embedding spatial and channel attention modules. These modules highlight the interdependent channel maps and focus on the discriminant regions, adaptively emphasizing the relevant features and neglecting the irrelevant information. The authors also proposed aggregation approaches to integrate learned attention with raw feature maps, further enhancing the network’s ability to highlight the salient features and suppress noise. Valanarasu et al. [25] introduced a gated axial attention model, extending the existing architectures with an additional control mechanism in the self-attention module. This gating mechanism prioritizes relevant features during the attention process. Additionally, the authors proposed a local–global training strategy for medical images, operating on whole images to capture the global features and on patches for the local features, thereby improving the model’s overall performance. Despite their advancements, the existing gated mechanisms did not incorporate a unified gated attention control mechanism combining channel-, spatial-, and element-wise attention to modulate feature flow.

### 2.4. Attention Mechanisms 

Woo et al. [26] proposed the convolutional block attention module (CBAM). The CBAM sequentially infers attention maps along two dimensions: channel and spatial. The channel includes both global average pooling and global max pooling, followed by shared multi-layered perceptron (MLP), while the spatial attention module applies convolution over concatenated average-pooled and max-pooled features along the channel axis. Hu et al. [27] proposed the squeeze and excitation (SE) attention block. SE attention adaptively recalibrates the channel-wise feature responses by explicitly modeling interdependencies between channels. The attention mechanism involves the squeeze block having a global average pooling to generate channel-wise statistics and the excitation block containing a fully connected layer followed by non-linearity (ReLU) and another fully connected layer filled by a sigmoid function. This generates weights for each channel. Recalibration is achieved by channel-wise multiplication of the original feature map with the generated weights. While attention mechanisms like CBAM and SE blocks improve feature representation, they are limited to static attention strategies and do not leverage dynamic control mechanisms. Furthermore, these modules do not integrate features at multiple scales, restricting their ability to handle multi-resolution data effectively.

### 2.5. Graph-Based Hybrid Models

Matlock et al. [28] introduced wave networks to address the limitation of the traditional graph convolutional networks (GCNs) in propagating long-range information across graphs. The authors demonstrated the superiority of wave networks over the traditional GCNs across three tasks: labelling the paths in graphs, solving mazes, and computing the voltages in circuits. The core idea was propagating information in waves across the graph via a breadth-first search, which allowed for more efficient long-range information propagation. Though the wave networks achieved a good performance, while requiring fewer parameters and computational resources than the traditional GCNs, the spectral computations required for wave networks can be computationally intensive, especially for large graphs. This limits their scalability to high-dimensional data, such as large-scale medical image datasets. Dong et al. [29] proposed a Dual-GCN framework for image captioning that integrates an object-level GCN and an image-level GCN. The object-level GCN extracts the spatial relationships between objects within an image, while the image-level GCN utilizes the similarities among multiple images to enhance global feature representation. These embeddings were combined and passed to a transformer-based linguistic decoder, enabling detailed and accurate image captioning. Additionally, the authors introduced a curriculum learning strategy to train the model by progressively incorporating more complex data samples, enhancing robustness and generalization. The Dual-GCN framework explicitly uses graph structures to model relationships, which is powerful, but computationally intensive, especially when generating global embedding from similar images due to the high computational demands of graph construction and similarity calculations.

## 3. Methods

### 3.1. Model Architecture

**Figure 1:**
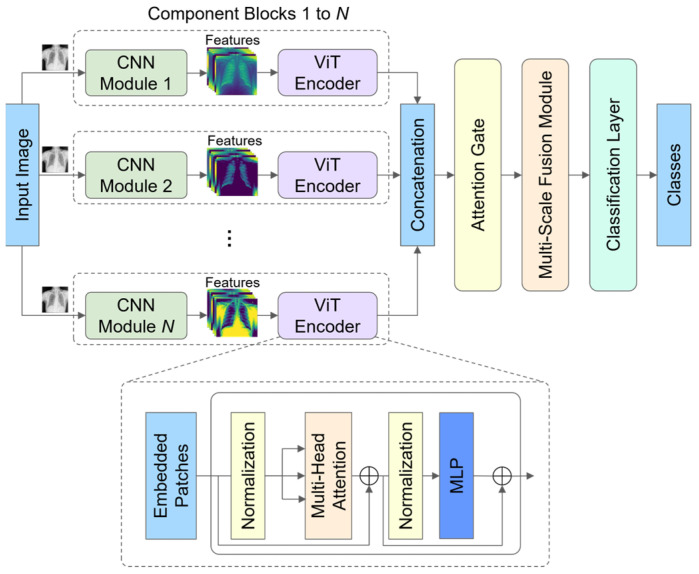
The proposed hybrid architecture.

The proposed model architecture, as shown in Figure 1, was designed to leverage the strengths of CNNs and the ViT for image classification. The architecture consists of multiple CNN-ViT component blocks, an attention gate mechanism, a multi-scale fusion module, and a classification layer. The architecture is modular, flexible, and aims to extract robust multi-scale features, while allowing for feature selection and fusion.

### 3.2. Component Blocks

Each component block begins with a base CNN model designed to extract low-level spatial features. The input image of a shape $\left(H,\;W,\;C\right)$ (height, width, and channel) is passed to one of the CNN models to extract feature maps. Afterward, the extracted feature maps are further reshaped to $\left(\frac{H}{P},\;\frac{W}{P},\;C\right)$ sequential patches (with a patch size P) with position embedding, making the shape compatible with the ViT encoder. The proposed architecture allows for flexibility in the number of component blocks, and each block can use a different CNN architecture depending on the requirements and available computational resources. Then, the sequential patches are then passed to the ViT encoder to capture long-range dependencies and global context information. The ViT encoder consists of a sequence of transformer encoder layers, each containing layer normalization, which standardizes the feature map inputs, reducing internal covariate shifts and improving the stability of the learning process. The normalized features are then passed through a multi-head self-attention mechanism, where the input sequence is split across multiple attention heads, each learning unique dependencies. For each head, the input features are projected into query, key, and value vectors, which are used to compute attention scores. Following the MHA, another normalization layer and then the multi-layer perceptron (MLP) layer are applied. The MLP layer comprises two fully connected layers with Gaussian Error Linear Unit (GELU), which introduces non-linearity and further enhances feature representation. To prevent overfitting, dropout layers are applied after each fully connected layer. Finally, residual connections are added around both the MHA and MLP layers. These connections help retain the original feature information, support gradient flow, and prevent the degradation of performance over multiple layers.

### 3.3. Gated Mechanism with Attention

**Figure 2:**
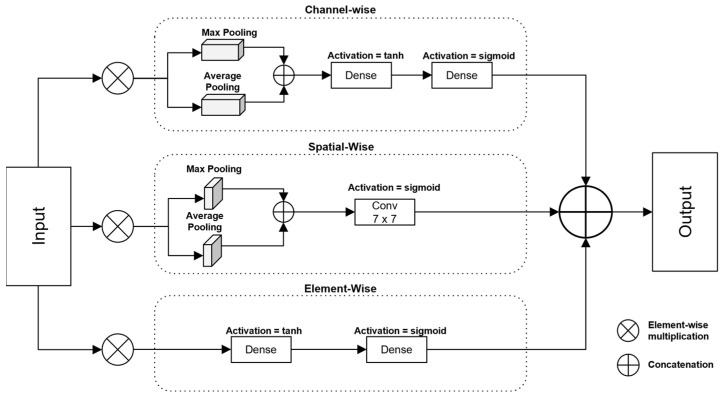
Gated mechanism with attention.

The gated mechanism (Figure 2) in the proposed architecture refines and controls the flow of important features extracted from the component blocks. It integrates channel attention, element-wise attention, and spatial attention mechanisms, each of which captures different aspects of the input feature to ensure that only the most relevant information is passed forward. These attention mechanisms operate independently, and their results are concatenated to create a comprehensive and enhanced feature representation.

#### 3.3.1. Channel-Wise Attention

The channel attention mechanism leverages global average pooling (*AvgPool*) and global max pooling (*MaxPool*) to highlight the most important channels in the input feature map X. The pooling operations summarize the feature information across all spatial locations for each channel. After pooling, the concatenated (*Concat*) feature map is passed through two fully connected layers, first with *tanh* activation, and then *sigmoid* (σ) activation, generating attention weights that emphasize the significant channels. These weights are then broadcast across the spatial locations, allowing for the model to scale each channel’s feature map by its importance.

Mathematically, the channel attention is computed as follows:

(1) $$A_{channel}=\sigma ({Dense}_{2}(tan\;h({Dense}_{1}(Concat\left(AvgPool(X\right),\;MaxPool(X))))))$$

$A_{channel}$ is applied to the input feature map through element-wise multiplication, highlighting the important channels.

#### 3.3.2. Element-Wise Attention

Element-wise attention is applied directly to each feature vector in the input feature map X. This mechanism generates attention scores for individual features within each spatial location. These scores are computed using two fully connected layers with *tanh* and *sigmoid* activations, like the channel attention mechanism. Element-wise attention generates attention scores by first projecting the feature map into a lower-dimensional space using linear transformation. Tanh activation emphasizes the most significant relationships by mapping the feature values within a symmetric range of [−1, 1]. This output is further scaled to [0, 1] using the sigmoid activation, which provides a probabilistic interpretation for the attention scores. This approach allows for the model to selectively emphasize or suppress specific elements in each feature vector.

(2) $$A_{element}=\sigma {(Dense}_{2}(tan\;h({Dense}_{1}(W_{e}X+b_{e}))))$$

The input feature map X is first linearly transformed using a weight matrix $W_{e}$ and bias $b_{e}$, capturing the relationships between the feature elements. The transformed features are then passed through tanh, which introduces non-linearity and ensures the output lies in the range [−1, 1]. sigmoid is applied to scale the values to [0, 1], making them suitable as attention weights. Element-wise attention is then applied via element-wise multiplication to the input feature map for high-level control of feature importance.

#### 3.3.3. Spatial-Wise Attention

Spatial attention captures the relationships between different spatial regions in the input feature map by reshaping it into its original 2D spatial form. The input feature map is reshaped, and global average pooling and global max pooling are applied across the channel dimension. The resulting pooled features are concatenated and processed by a 7 × 7 convolutional (Conv) layer with *sigmoid* activation to generate spatial attention weights. These weights are applied to the feature map to emphasize the important spatial regions.

(3) $$A_{channel}=\sigma (Conv(Concat\left(AvgPool(X\right),\;MaxPool(X))))\;\;$$

The attended spatial features are then reshaped back into their original form and multiplied element-wise with the input feature map.

#### 3.3.4. Combining Attention

Finally, the outputs of the channel attention, element-wise attention, and spatial attention mechanisms are concatenated along the feature dimension to form the final, enhanced feature representation. The combined attention map ensures that the most significant channels, elements, and spatial regions are retained, enabling the model to emphasize the critical features, while suppressing less-relevant information.

### 3.4. Multi-Scale Fusion Module

**Figure 3:**
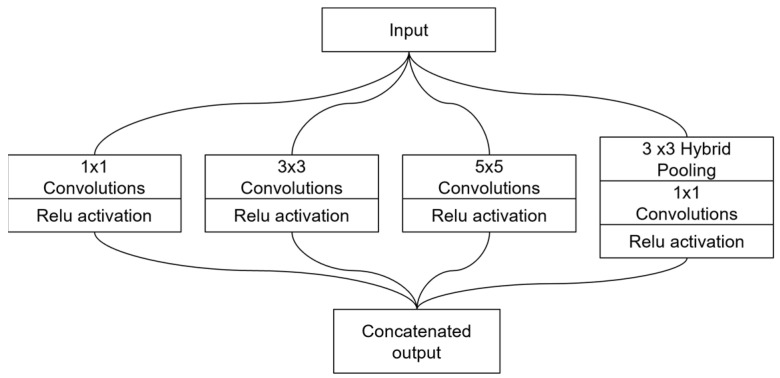
Inception-styled multi-scale fusion module proposed in this study.

The multi-scale fusion module in Figure 3 was designed to fuse multi-scale features by using an Inception [30] module-style architecture. This module consists of several branches to capture information at different scales. Branch 1 uses 1 × 1 convolution to capture fine-grained and localized details from the feature maps. These operations reduce dimensionality and mitigate the computational cost. Branch 2 uses 3 × 3 convolution to capture medium-scale features, effectively balancing spatial resolution and contextual information. Branch 3 uses 5 × 5 convolution to capture broader and larger-scale features, which is crucial for identifying the patterns spanning larger regions in the image. Branch 4 applies hybrid pooling [31], a combination of max pooling and Hartley spectral pooling [32], by 1 × 1 convolution to capture and preserve very large-scale features and the global context. The Harley pooling technique transforms the input feature maps into a frequency domain using discrete Hartley transform. By operating in the frequency domain, Harley spectral pooling captures the global spatial structures, while filtering out high-frequency noises. This method retains more spatial information than max pooling, which alleviates the resolution loss issue inherent in max pooling by preserving more spatial information. In each of the branches, the ReLU activation function was applied to the convolution layers. These branches were concatenated along the channel axis, resulting in comprehensive feature representation across the different scales.

### 3.5. Classification Layer

After the multi-scale fusion module, the output features were flattened and passed through two fully connected layers. Dropout layers were used to prevent overfitting during training. The final fully connected layer employed the SoftMax activation function to produce class probabilities, corresponding to the number of target classes for the classification tasks in this study.

## 4. Experimental Results and Discussion

### 4.1. Dataset Description

The dataset used consists of postal-view X-ray images, which were sourced from the publicly available databases described as follows:

1. TB class: TBX11k [33], the National Library of Medicine (NLM) dataset [34], the NIAID TB dataset [35], and the Diagnox and PRORAD URS datasets [36].
2. COVID-19 class: the COVIDx-CXR-3 dataset [37] and the Extensive COVID-19 X-Ray and CT Chest Images dataset [38].
3. Pneumonia class: the Pneumonia dataset [39] and the RSNA Pneumonia Dataset [40]
4. Normal class: the normal class was assembled from the various datasets mentioned above.

For the training and evaluation of the proposed model, a combined dataset was used for each class to ensure more diverse and representative sample images, which contributed to the robustness and generalizability of the trained model. Additionally, pixel-to-pixel comparison was conducted to ensure no redundancy or repetition of images when combining the datasets. The combined dataset may have introduced biases due to the differences in annotation protocols across repositories, which may lead to variable image quality and inconsistent labels, impacting the model’s ability to generalize to unseen data. To mitigate these biases, we employed pixel normalization, which scales the pixel values of an image to a range of [0, 1] by dividing the pixel values by 255 (the maximum intensity value for an 8-bit image). The custom dataset comprises 19,621 samples for the COVID-19 class, 17,952 samples for healthy people, 10,285 samples for the Pneumonia class, and 2851 samples for the TB class. To enhance the model’s performance and prevent overfitting because of the data imbalance, we applied data augmentation during the data processing stage, as described in Section 4.5.

### 4.2. Experimental Parameters and Environment

For the experiments, we used two CNN-ViT component blocks, consisting of two base CNNs, EfficientNetB3 [41] and DenseNet-121 [42], respectively. We achieved the best results when we set all the CNN layers to be trainable and started with pre-trained weights, which helped leverage prior knowledge for better feature extraction. The hyperparameter configurations of the Vitt encoder are summarized in Table 1. The choice of six Vitt encoder layers and two multi-head self-attention blocks strikes a balance between capturing long-range dependencies and maintaining computational efficiency. The hidden dimension of 32 and the MLP dimension of 64 ensure compact, yet expression representations, which help prevent overfitting. A dropout rate of 0.5 was applied consistently to mitigate overfitting by randomly disabling the neurons during training. A patch size of eight and an image resolution of 224 × 224 pixels were selected to preserve the spatial details. In the classification layer, we used dense units of 1024 and 128 for the first and second layers, respectively, with a dropout rate of 0.5 after each layer. This configuration effectively balances feature dimensionality reduction with enhanced discriminative power. In the classification layer, we used dense units of 1024 and 128 for the first and second layers, respectively, with a dropout rate of 0.5 after each layer. SoftMax activation was used in the classification layer as it is suitable for multi-class classification. When compiling the model, we used the Adam optimization and a categorical cross-entropy loss. For the learning rate, we set its initial value as 1 × 10^−5^, and then applied *ReduceLROnPlateau* callback (factor = 0.1, patience = 5) from *keras* to decrease the learning rate to enhance the convergence of the model. The experiments were performed on a Nvidia RTX 3090 GPU (NVIDIA Corporation, Santa Clara, CA, USA).

### 4.3. Evaluation Metrics

The performance of our proposed model was evaluated using the following evaluation metrics:

Accuracy represents the ratio of correctly classified cases to the total number of cases:

(4) $$Accuracy=\frac{TP+TN}{TP+TN+FP+FN}\;$$

where true positives (TPs) represent the number of cases correctly classified into the class they belong to, and true negatives (TNs) represent the number of cases correctly classified as not corresponding to a class. False positives (FPs) represent the number of cases incorrectly classified into a class, and false negatives (FNs) represent the number of cases incorrectly classified as not belonging to a class.

Precision measures the ratio of positive, correctly predicted cases to the total number of positive classification predictions [43]:

(5) $$Precision=\frac{TP}{TP+FP}\;$$

Recall measures the ratio of the actual positive, correctly predicted cases:

(6) $$Recall=\frac{TP}{TP+FN}\;$$

The F1-score measures the average of precision and recall [41].

(7) $$F1=2\times \frac{Precision\times Recall}{Precision+Recall}\;$$

### 4.4. Classification Results of the Proposed Model

**Figure 4:**
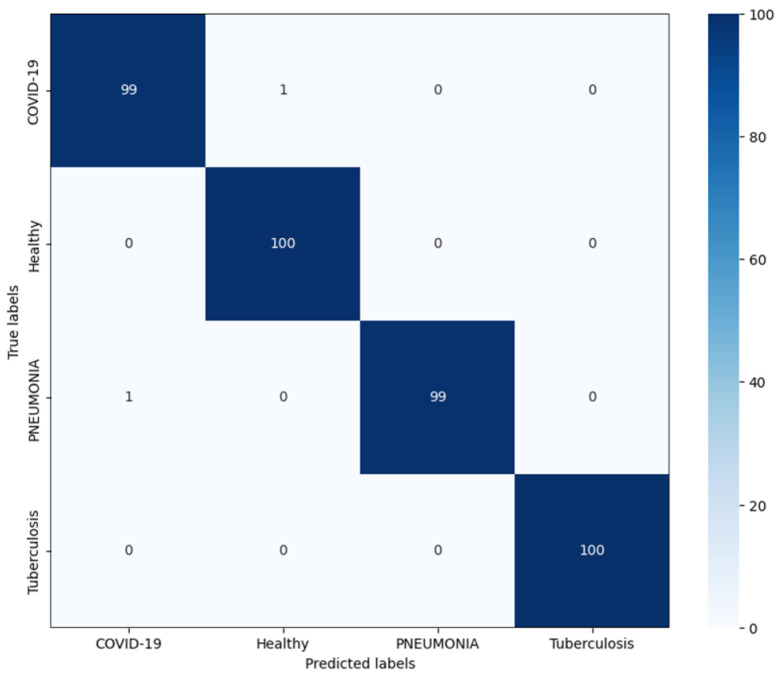
A confusion matrix for the proposed model.

The performance of the proposed model is shown in Table 2. The model achieves an overall accuracy of 99.5% on the multi-class classification task when evaluated on the test set, with an individual class accuracy of 99.0% for the COVID-19 class, 100.0% for the healthy samples, 99.0% for the Pneumonia class, and 100.0% for the TB class. The precision, recall, and F1-score for each class are ranged from 0.99 to 1.00. The high performance across all the classes demonstrates the effectiveness of the proposed model in accurately classifying various types of abnormalities in medical imaging. In Figure 4, the confusion matrix provides a detailed view of the classification performance of the proposed model across the four classes. For the COVID-19 and Pneumonia classes, 99 out of 100 samples were correctly classified, with only 1 sample being misclassified for each. This slight misclassification may be caused by the overlapping features between the COVID-19 and Pneumonia chest radiographs, potentially due to cases with the infection of COVID-19 and Pneumonia at the same time. The healthy and TB classes show perfect classification, with 100 correct predictions for each. The ability to correctly classify most class samples indicates that the model effectively captures the unique patterns associated with these diseases.

### 4.5. Ablation Studies

**Figure 5:**
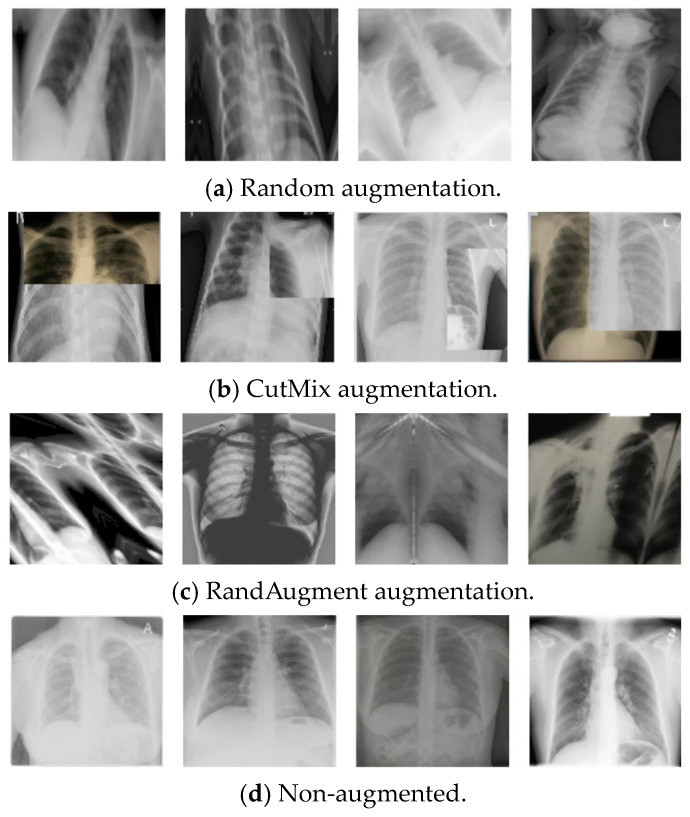
Impact of different augmentation methods on original images.

Table 3 presents the results of our investigation on the impact of various data augmentation techniques on the performance of the model. In our experiment, we observed that CutMix [44] and RandAugment [45] are effective in improving the model’s performance, achieving an accuracy of 99.50%. As illustrated in Figure 5, CutMix involves combining patches from two images and mixing their labels proportionally to the area of the patches, which enables the model to focus on the less-discriminative parts of an object. RandAugment, on the other hand, applies a fixed number of randomly chosen transformations with adjustable magnitudes. The model without any data augmentation achieves an accuracy of 98.25%.

**Figure 6:**
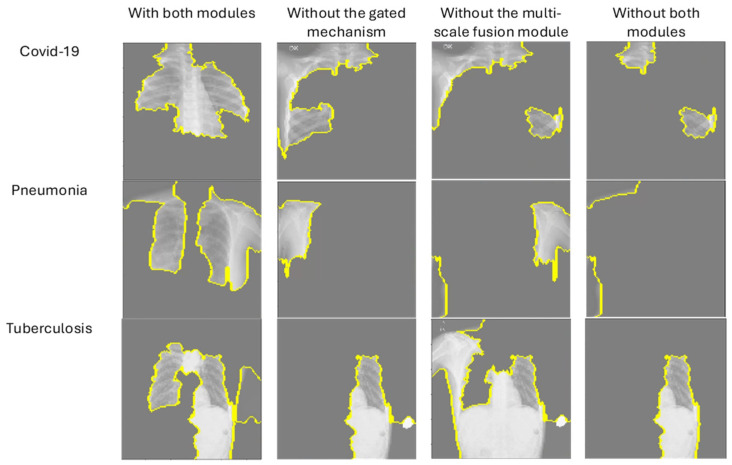
Impact of gated mechanism and multi-scale fusion using LIME explainability analysis.

To further investigate the effectiveness of the proposed components, we analyzed the impact of the gated mechanism and the multi-scale fusion module on the overall performance of the model. Table 4 presents the results of this ablation study. When the gated mechanism was removed from the model, the accuracy dropped to 99.25%, indicating that the gated mechanism plays a crucial role in enabling the model to effectively control and select important features extracted from the CNN and ViT components. Similarly, removing the multi-scale fusion module resulted in a decrease in accuracy to 99.00%, indicating the importance of the multi-scale fusion module in enabling the model to capture features at multiple scales and improve the overall classification performance. Lastly, when both the gated mechanism and the multi-scale fusion module were added, the accuracy was 99.50%, indicating the positive effect of these proposed components in enhancing the model’s performance. Figure 6 illustrates the effectiveness of the proposed model using LIME [46] explainability analysis. The highlighted regions in the images (outlined in yellow) correspond to the most important areas identified by the model for classification. The figure showcases results across various samples, with the clear localization of critical regions relevant to the classification task. These visualizations confirm that the model successfully focuses on pertinent features, such as abnormal regions in chest X-rays, reinforcing the significance of the gated mechanism and the multi-scale fusion module in driving the attention towards diagnostically relevant areas.

Next, we examined the impact of increasing the number of component blocks in our model architecture, as shown in Table 5. This experiment clearly demonstrates that as the number of component blocks increases, the accuracy improves significantly. Starting with a single component block (CNN model: EfficientNetB3 [41]), the model achieves an accuracy of 96.50%. With two component blocks (CNN models: EfficientNetB3 and DenseNet-121 [42]), the classification accuracy increases to 99.50%. With three component blocks (CNN models: EfficientNetB3, DenseNet121, and MobileNet [47]), the accuracy slightly increases to 99.55%. This indicates that the inclusion of additional component blocks allows for the model to capture more diverse and complex features. The key contributor to performance improvement as the number of component blocks increases is the use of different CNN models in each component block. By employing a variety of CNN architectures, each block can learn distinct representations from the input data. This approach introduces complementary features from different CNNs, enhancing the model’s overall capacity to generalize well across various data patterns.

### 4.6. Comparison with Existing Models

The proposed CNN-ViT model achieves a classification accuracy of 99.50%, outperforming the existing hybrid approaches listed in Table 6. This performance improvement can be attributed to the integration of gated attention mechanisms and multi-scale fusion, which enable the effective combination of local and global features, addressing challenges like long-range dependencies and contextual learning. Despite its high performance, our model has inherent limitations. The reliance on extensively labeled datasets may limit its generalizability, particularly in scenarios with data scarcity or varying annotation standards. These limitations underscore the need for future work exploring self-supervised learning (SSL) to reduce the dependency on labeled data, while maintaining accuracy.

## 5. Conclusions

The results of this study demonstrate that the proposed CNN-ViT hybrid model, incorporating a gated mechanism with attention and a multi-scale fusion module, outperforms the state-of-the-art studies in pulmonary disease classification. Achieving an accuracy of 99.50%, the model’s superior performance is attributed to the seamless integration of CNN and ViT encoder layers, which balances local feature extraction with long-range dependency capture. This hybrid approach addresses the individual limitations of CNN and ViT architectures by effectively combining the CNN’s strength in capturing local features with the ViT’s capacity for global feature representation. The success of this model suggests that a hybrid CNN-ViT architecture with enhanced feature selection can serve as a robust solution for complex, multi-class medical image classification tasks.

The proposed model uses a different CNN architecture in different component blocks, such as EfficientNetB3 and DenseNet-121, for feature extraction. EfficientNetB3 captures the features efficiently with fewer parameters, while DenseNet-121 focuses on feature reuse through densely connected layers, enhancing representation learning. Feeding the output of these CNN architectures to the ViT allows for the model to leverage various feature maps from different CNN architectures and utilize the strength of the ViT to capture long-range dependencies across these features to learn the subtle disease patterns in medical images.

The broader impacts of this research can be extended to real-world applications, particularly in resource-constrained clinical settings. The model’s efficiency and high accuracy make it suitable for the rapid diagnosis of pulmonary diseases, which is critical for early detection and treatment in underserved areas. Additionally, this framework can be adapted for other medical imaging tasks, providing a generalized approach for disease diagnosis.

To ensure repeatability of the proposed method, we conducted all the experiments multiple times under consistent conditions, including random seed initialization, fixed dataset splits, and the hyperparameter configurations. The results reported represent the average performance of our model across different runs, ensuring the reproducibility and robustness of the findings.

A significant challenge to this research is the availability of labeled medical imaging data, as annotating large datasets is labor-intensive and requires domain expertise. We plan to address this limitation by leveraging SSL techniques, such as SimCLR [51] (Simple Contrastive Learning of Representation) and BYOL [52] (Bootstrap Your Own Latent). We will implement the SSL technique using a teacher–student concept, where a teacher model performs a pre-text task to extract meaningful features, such as predicting the image rotations, the patch orders, or colorization, and a student model learns from these features for downstream tasks like classification. By using SSL, we aim to reduce the dependency on labeled data, while enabling the model to achieve competitive accuracy.

Furthermore, reducing model complexity remains a priority, as attention mechanisms like MHA add considerable computational demands. In future work, exploring attention mechanisms that lower the computational load, such as Linformer [53], Performer [54], or ProbSparse [55] attention, could streamline the architecture without sacrificing the model’s performance. Additionally, we observed that the model tends to confuse some images between the Pneumonia and COVID-19 classes. This may be due to the fact that COVID-19 patients are often complicated with Pneumonia [56], leading to overlapping features in chest X-ray images. The datasets used for training and evaluation were sourced from different repositories, increasing the likelihood that some images labeled as COVID-19 might also exhibit signs of Pneumonia. Refining the model to address these misclassifications will also involve curating more refined datasets that emphasize subtle distinctions between these classes or applying transfer learning with domain-specific datasets to enhance the model’s ability to distinguish overlapping visual features. Such improvements would enhance the model’s practicality in clinical settings, advancing its applicability to a broader range of diagnostic challenges.

Lastly, despite the strong performance of the proposed model, one limitation is that it was not calibrated during this study. Model calibration is crucial, particularly in clinical applications, as it ensures that the predicted probabilities are reflective of the true likelihood of correctness. Calibration addresses the issue of overconfidence, where the model’s high-level confidence in predictions does not always correspond to the correct prediction. In future work, we aim to incorporate temperature scaling, a post hoc calibration technique to align the predicted probabilities with actual accuracy to not only bolster the reliability of the model’s predictions, but also strengthen its suitability for practical deployment in clinical settings.

## Figures and Tables

**Table 1:** Hyperparameter configurations of ViT encoder.

Hyperparameters	Value
Number of ViT encoder layers	6
Hidden dimension	32
Multi-layer perceptron dimension	64
Number of multi-head self-attention blocks	2
Dropout rate	0.5
Patch size	8
Image channels	3
Image size	224 × 224
Epoch	51

**Table 2:** Classification results of proposed model.

Category	Precision	Recall	F1-Score	Accuracy	Overall Accuracy
COVID-19	0.99	0.99	0.99	99.0%	99.5%
Healthy	0.99	1.00	1.00	100.0%	
Pneumonia	1.00	0.99	0.99	99.0%	
Tuberculosis	1.00	1.00	1.00	100.0%	

**Table 3:** Impact of data augmentation on classification accuracy.

Data Augmentation	Classification Accuracy
CutMix [44]	99.50%
RandAugment [45]	99.50%
Without augmentation	98.25%

**Table 4:** Impact of gated mechanism and multi-scale fusion module on classification accuracy.

Models	Classification Accuracy
Without the gated mechanism	99.25%
Without the multi-scale fusion module	99.00%
With both modules	99.50%

**Table 5:** Impact of increasing number of component blocks on classification accuracy.

Models	Classification Accuracy
1 component block (CNN model: EfficientNetB3 [41])	96.50%
2 component blocks (CNN models: EfficientNetB3 and DenseNet-121 [42])	99.50%
3 component blocks (CNN models: EfficientNetB3, DenseNet-121, and MobileNet [47])	99.55%

**Table 6:** Comparison with existing hybrid models on multi-class classification tasks.

Studies	Classification Accuracy
Barhoumi and Rasool [48]	98.04%
Chen et al. [49]	96.60%
Shah et al. [50]	90.00%
The proposed model	99.50%

## Data Availability

The raw data supporting the conclusions of this article will be made available by the authors on request.
